# Screening for insecticide resistance in Australian field populations of *Bemisia tabaci* (Hemiptera: Aleyrodidae) using bioassays and DNA sequencing

**DOI:** 10.1002/ps.6906

**Published:** 2022-06-03

**Authors:** Cao Fang, Jamie E Hopkinson, Jacob Balzer, Michael Frese, Wee Tek Tay, Thomas Walsh

**Affiliations:** ^1^ Faculty of Science and Technology University of Canberra Canberra Australia; ^2^ CSIRO Acton; ^3^ Department of Agriculture and Fisheries Queensland Government Toowoomba Australia; ^4^ Department of Applied BioSciences Macquarie University Sydney

**Keywords:** whitefly, insecticide resistance, pyrethroid, organophosphate, pest management, surveillance

## Abstract

**Background:**

Species within the *Bemisia tabaci* cryptic species complex can cause significant crop damage. We used high‐throughput amplicon sequencing to identify the species composition and resistance allele genotypes in field populations from cotton fields in Australia. For selected populations, the resistance phenotype was determined in bioassays and compared with sequencing data.

**Results:**

A metabarcoding approach was used to analyse the species composition in 144 field populations collected between 2013 and 2021. Two mixed AUS I and MEAM1 populations were detected, whereas the remaining 142 populations consisted of MEAM1 only. High‐throughput sequencing of organophosphate and pyrethroid resistance gene amplicons showed that the organophosphate resistance allele F331W was fixed (> 99%) in all MEAM1 populations, whereas the pyrethroid resistance allele L925I in the voltage‐gated sodium channel gene was detected at varying frequencies [1.0%–7.0% (43 populations); 27.7% and 42.1% (two populations); 95%–97.5% (three populations)]. Neither organophosphate nor pyrethroid resistance alleles were detected in the AUS I populations. Pyrethroid bioassays of 85 MEAM1 field‐derived populations detected no resistance in 51 populations, whereas 32 populations showed low frequency resistance, and 2 populations were highly resistant.

**Conclusions:**

We demonstrate that high‐throughput sequencing and bioassays are complementary approaches. The detection of target site mutations and the phenotypic provides a comprehensive analysis of the low‐level resistance to pyrethroids that is present in Australian cotton farms. By contrast, a limited survey of whitefly populations from horticulture found evidence of high‐level resistance against pyrethroids. Furthermore, we found that the F331W allele (linked to organophosphate resistance) is ubiquitous in Australian MEAM1. © 2022 Commonwealth of Australia. *Pest Management Science* published by John Wiley & Sons Ltd on behalf of Society of Chemical Industry.

## Graphical abstract

Molecular and bioassay assessments of organophosphate and pyrethroid resistance in whitefly populations show that organophosphate resistance alleles are fixed but pyrethroid resistance alleles are present at low frequencies in the Australian cotton system.

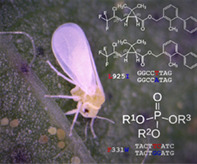

## 1 INTRODUCTION

*Bemisia tabaci* ^1^ (Insecta: Hemiptera: Aleyrodidae), commonly called tobacco whitefly, cotton whitefly or silverleaf whitefly, consists of a complex of phloem‐feeding and morphologically indistinguishable insects. *B. tabaci* is highly polyphagous, capable of feeding on a wide range of agricultural and horticultural crops, and infestations often result in significant economic losses around the world (for example, an estimated US$5 billion loss due to cotton leaf curl disease in Pakistan between 1992 and 1997^2^ and an estimated US$3 billion loss to the Brazilian agriculture between 1995 and 2019^3^). The insects harm plants by feeding on phloem sap and excreting ‘honeydew’, a sugar liquid that encourages the growth of sooty mould,^4^ which significantly reduces photosynthesis.^5^ Honeydew is of particular concern for cotton growers because it leads to ‘sticky cotton’, causing problems in cotton gins and textile mills.^6^, ^7^ *B. tabaci* is also an effective vector for plant viruses.^8^ For example, *B. tabaci* transmits the *Cotton leaf curl virus*, the etiological agent of cotton leaf curl disease, the *Tomato yellow leaf curl virus*, one of the most important tomato pathogens,^6^, ^8^ and the *Bean golden mosaic virus* that infect crops such as cucurbits and soybeans.^8^, ^9^

*B. tabaci* was originally thought to be a single species but is now recognised as a cryptic species complex.^10^, ^11^ Genetic differences allow for a reliable species identification through partial sequencing of the mitochondrial cytochrome *c* oxidase subunit I (mtCOI) gene.^11^, ^12^, ^13^, ^14^ Within the *B. tabaci* complex, two species have been recognised as highly invasive, Middle East–Asia Minor 1 (MEAM1) and Mediterranean (MED), previously known as *B. tabaci* biotypes B and Q, respectively.^15^ Both MEAM1 and MED are polyphagous and highly fecund, and in most places, have evolved resistance to commonly applied insecticides.^6^, ^9^

Around the world, MEAM1 has evolved resistance to many widely used pesticides, including pyrethroids and organophosphates (OPs).^6^, ^16^, ^17^ Resistance against pyrethroids and OPs is caused by changes to the metabolic activity (for example, through the detoxification of pesticides)^18^ and/or target site insensitivity.^17^, ^19^ Metabolic resistance generally involves hydrolytic and oxidative pathways^18^, ^20^ and pyrethroid resistance in MEAM1 has been associated with increased ester hydrolysis.^18^ OP resistance can be conferred by esterase‐based metabolic resistance that involves sequestration or degradation of the pesticides; in MEAM1, an elevated activity of carboxylesterase is associated with OP resistance.^17^, ^21^

Target site resistance to pyrethroids and OPs is linked to mutations in the voltage‐gated sodium channel gene (*vgsc*) and the acetylcholinesterase gene (*ace1*), respectively. Pyrethroids exert their toxic effects by binding and stabilising the open state of the voltage‐gated sodium channel,^22^, ^23^ which leads to persistent membrane depolarisation and hyperexcitability, causing paralysis and death of insects.^24^ Resistance to pyrethroids can be conferred by point mutations close to a hydrophobic pyrethroid‐binding site (for example, L925I and T929V).^25^, ^26^ In insects, OPs target the enzyme acetylcholinesterase (AChE) by phosphorylating the active site serine of AChE, which permanently inactivates the enzyme,^27^, ^28^ results in build‐up of acetylcholine, and lead to insect paralysis and death. Mutations in AChE (for example, F331W) are associated with OP resistance in MED^16^ and MEAM1.^29^ How F331W causes resistance is not completely understood, but because position 331 is located close to the active site,^30^ the change to tryptophan may have steric effects that protect the enzyme from interacting with OPs.

MEAM1 was detected in Australia in 1994^31^ and is now widely distributed across the mainland of Australia. Of the other invasive *B. tabaci* cryptic species, Asia II was recently detected in Australia^32^ and MED has not been found and is considered absent.^32^ Furthermore, Australia has two endemic *B. tabaci* species, AUS I formerly known as Eastern Australian native (EAN) and AUS II formerly known as Western Australian native.^33^, ^34^ The study that reported the arrival of MEAM1 in Australia, also documented the appearance of AChE‐mediated resistance to OPs and carbamates. More recent studies detected resistance to pyrethroids, pyriproxyfen^29^ and spirotetramat.^35^

Since the arrival of MEAM1 in Australia, insecticide resistance in the field has been monitored by state agriculture departments. The first major outbreak of MEAM1 in cotton was observed near Emerald, central Queensland (Qld) during the summer of 2001/2002, which triggered the development of a pest management plan for MEAM1.^36^ In a previous study, we reported on the field resistance of MEAM1 against various pesticides (for example, pyriproxyfen and bifenthrin) using a bioassay.^29^ In this study, we used metabarcoding and high‐throughput sequencing (HTS) to determine the species composition and resistance gene frequencies to pyrethroids and OPs in *B. tabaci* field samples from New South Wales (NSW) and Qld between 2013 and 2021. Furthermore, we used pyrethroid bioassay data from the same period to test for a correlation between the frequency of the resistance allele for pyrethroid resistance and survival at the discriminating dose.

## 2 MATERIALS AND METHODS

### 2.1 Sample collections, rearing and lab strains

**FIGURE 1:**
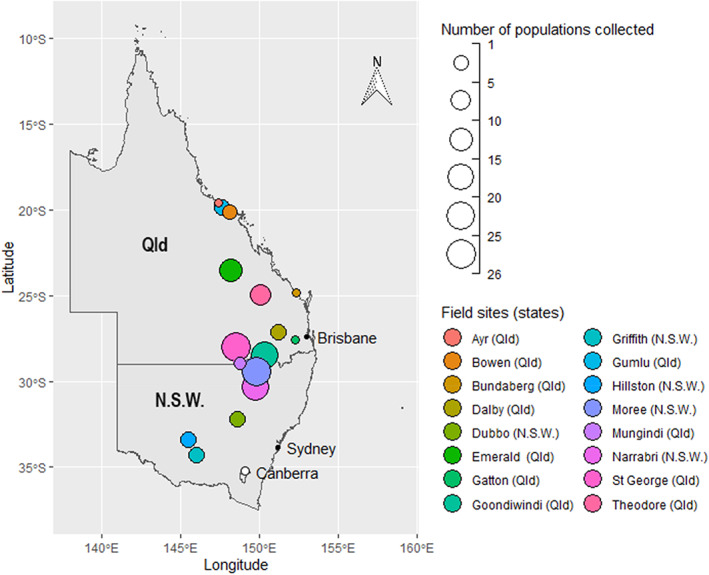
*Bemisia tabaci* collection sites from Queensland (Qld) and New South Wales (N.S.W.). Circle sizes represent the number of population samples collected. Different field sites are represented by different colours, for example 25 populations were collected from Moree.

Whitefly (*B. tabaci* species complex) populations (*n* = 144) were collected between 2013 and 2021 from agricultural crops, primarily cotton (during late boll filling) in NSW and Qld (Figure 1, Table 1 and Table S1). Adult whiteflies were collected from crops using a petrol‐powered vacuum (Stihl BG75) fitted with a gauze collecting sock, then transferred into cages with plant material and transported to the laboratory. In a small number of cases, leaves infested with whitefly nymphs were collected from crops by agronomists and sent by courier service to the laboratory. Upon arrival at the laboratory, each population was transferred to a rearing cage (63 × 35 × 61 cm) containing an insect‐free cotton plant, enabling adult whiteflies to be separated from any predators, parasitoids or cotton pests that could potentially harm the establishment of breeding colonies.^29^ These caged insect populations were kept in a glasshouse [25°C, 60% relative humidity (RH)] and reared for several generations. Cotton plants (*Gossypium hirsutum*, varieties Sicot 71BRF and Sicot 714B3F) were used in bioassays and to maintain whitefly populations in the laboratory. Plants were grown in pots (containing a blend of potting mix, perlite sand and fertiliser) under artificial light in controlled‐environment rooms (29°C, 70% RH, 16:8 h light/dark photoperiod) for 3 weeks and then moved to large insect‐proof cages in which they continued to grow under glasshouse conditions (25°C, 60% RH) for a further 3 weeks.

**TABLE 1:** *Bemisia tabaci* sampling information

Field sample region^a^	Number of *B. tabaci* populations^b^	Year of sampling
Dalby	4	2015, 2017, 2019, 2020
Emerald	13	2013, 2015, 2016, 2017, 2018, 2019, 2020, 2021
St George	26	2013, 2015, 2016, 2017, 2018, 2019, 2020, 2021
Theodore	10	2013, 2015, 2016, 2017, 2018, 2019, 2020, 2021
Hillston	4	2016, 2017, 2020, 2021
Griffith	4	2015, 2018, 2019, 2021
Dubbo	4	2018, 2019, 2020, 2021
Bowen	3	2019
Narrabri	20	2013, 2015, 2016, 2017, 2018, 2019, 2020, 2021
Gatton	1	2013
Goondiwindi	20	2015, 2016, 2017, 2018, 2019, 2020, 2021
Mungindi	2	2016, 2019
Moree	25	2013, 2015, 2016, 2017, 2018, 2019, 2020, 2021
Gumlu	3	2019
Ayr	1	2016
Sampling information for *B. tabaci* laboratory strains		
Bundaberg (AN12‐1)	1	2012^c^
Gumlu (GU10‐1R)	1	2011^d^
Ayr (AY09‐1R)	1	2009^e^
SU07‐1	1	1995

^a^ Locations from which field populations were sampled.

^b^ Total number of whitefly populations collected in a given region.

^c^ The native AUS I population was collected from Bundaberg in 2012.

^d^ Population collected from Gumlu in 2011 was selected with pyrethroids to create a pyrethroid‐resistant laboratory strain.

^**e**^ Population collected from Ayr in 2009 was selected with pyriproxyfen.

This study includes a laboratory strain of *B. tabaci* MEAM1 (‘SU07‐1’) that is used as a pyrethroid susceptible control; this strain was established in Toowoomba in 2007 from a population that was collected in 1995 by CSIRO in Canberra, Australian Capital Territory. Since the time of collection, the population has had no exposure to insecticides but nevertheless has retained its resistance to OPs.^29^ A pyrethroid‐resistant MEAM1 population (‘GU10‐1R’) was collected near the town of Gumlu in north Qld in 2010 and selected for resistance with increasing doses of bifenthrin (2–30 g L^−1^); this population is maintained as a highly resistant reference population via selection with 1 g L^−1^ bifenthrin (once per generation).^29^ As a control for the identification of endemic Australian whitefly species, a laboratory population of AUS I (‘AN12‐1’) was established. This native population was collected in 2012 from whiteflies found on the invasive coastal weed *Euphorbia cyathophora* (painted spurge) in Bargara, Qld. During the establishment of the ‘AN12‐1’ populations, about 20 individuals from each generation were preserved in ethanol and the species status was determined using random amplification of polymorphic DNA (RAPD) polymerase chin reaction (PCR) of the BEM23 and OPH16 markers.^37^, ^38^

### 2.2 Dose–response bioassay

*B. tabaci* MEAM1 populations were screened for the presence of resistance to pyrethroids using formulated bifenthrin (for 2013–2019, with 250 g L^−1^ Astral Nufarm, and for 2020–2021, with 240 g L^−1^ Venom Adama). For each population, the bioassay was typically completed within one to four generations of laboratory breeding and only in rare cases, in later generations (Table S1). We used a leaf‐dip bioassay,^39^ modified by using clip cages on detached leaves instead of leaf discs in Petri dishes. Bifenthrin was diluted in deionised water with the additive Agral at 100 mg L^−1^: in 2013–2015, treatment doses ranged from 1–1000 mg L^−1^ (with some variation), and from 2016 onwards, 1, 10, 100, 320 and 1000 mg L^−1^ were used; a control treatment (diluent only) was included. Leaves were dipped into the insecticide solution for 20 s and then dried at room temperature (25°C) for 30 min. After drying, clip cages were attached to the leaves and adult whitefly were aspirated into each cage. The experiments were maintained in controlled‐environment rooms (25°C, 60% RH, 14:10 h light/dark photoperiod). Mortality was assessed at 48 h, with insects classified as alive if they showed any sign of movement.^39^ All treatment doses and the control were replicated five times, with 15–20 adult whiteflies in each experimental unit. Adult whiteflies surviving the discriminating dose of 300 mg L^−1^ bifenthrin, as determined in bifenthrin bioassays undertaken between 2010 and 2015 ^29^, were defined as resistant.

### 2.3 Sequencing of mtCOI, *ace1* and *vgsc* genes

Subsamples (*n* ~ 10–100) from field and laboratory whitefly populations were preserved in 90% ethanol and sent from the Department of Agriculture and Fisheries Laboratory in Toowoomba to the CSIRO Black Mountain Laboratory for molecular species diagnostics and a screening of resistance alleles.

Upon arrival, all shipped insect samples were stored at −20°C. DNA was extracted from single‐ or mixed‐sex pools of whiteflies in three replicates using the QIAGEN DNeasy Blood & Tissue kit following the manufacturer's instructions. Amplicons were generated using modified gene‐specific primers (Table 2), attached to Illumina linker sequences (Table 2, in bold). The linker sequences enable the addition of the Illumina barcodes [i5] and [i7] by a second round of PCR. The mtCOI barcoding region widely used in *B. tabaci* cryptic species identification^11^ was amplified using Wfly‐PCR‐F1/R1 and Wfly‐PCR‐F2/R2 primers (Table 2). The two sets of mtCOI primers amplified two overlapping contigs that cover the 657‐bp mtCOI barcode region.^14^ Primers Bt‐kdr‐F1 and Bt‐kdr RIntr1 were used to amplify 184 bp of the *vgsc* gene^16^; primers Bt‐ace‐F and Bt‐ace‐R were used to amplify 287 bp of the *ace1* gene.^16^

**TABLE 2:** Primer sequences used in this study

Primer name^a^	Primer sequence (excluding adapters)^b^	Amplicon size (excluding adapters)
mtCO1 gene‐specific primers		
Wfly‐PCR‐F1	TGGTTYTTTGGTCATCCRGAAG	645 bp
Wfly‐PCR‐R1	GGAAARAAWGTTAARTTWACTCC	
Wfly‐PCR‐F2	CGRGCTTAYTTYACTTCAGCYAC	663 bp
Wfly‐PCR‐R2	GGYTTATTRATTTTYCAYTCTA	
*ace1* gene‐specific primers		
Bt‐ace‐F	TAGGGATCTGCGACTTCCC	287 bp
Bt‐ace‐R	GTTCAGCCAGTCCGTGTACT	
*vgsc* gene‐specific primers		
Bt‐kdr‐F1	GCCAAATCCTGGCCAACT	184 bp
Bt‐kdr‐Rintr1	GAGACAAAAGTCCTGTAGC	

^a^ Specific primers for the partial amplification of mtCO1, *ace1* and *vgsc* genes.

^b^ Given gene‐specific primer sequences are attached to the linker sequences (in bold) when ordering which Illumina adapters [i5] and [i7] are attached to the linker sequences during the second amplification step 5′‐[i5]**TCGTCGGCAGCGTCAGATGTGTATAAGAGACAG**‐3′ and 5′‐[i7]**GTCTCGTGGGCTCGGAGATGTGTATAAGAGACAG**‐3′. ([i5] and [i7] are Nextera index sequences). Underlined sequences are recognition sites for trimming the adapter sequences during analysis steps (Illumina). Wfly‐PCR‐F1/R1 and Wfly‐PCR‐F2/R2 primers were the same as reported previously.^14^

All PCRs were performed using Platinum Taq (Invitrogen); mtCOI and resistance gene sequences were amplified as described.^14^, ^16^ If a population sample contained < 30 whiteflies, then three individual whiteflies were Sanger sequenced; if a sample contained > 30 whiteflies, pooled samples containing 10 or 20 whiteflies were used for metabarcoding and HTS.^14^, ^40^ Sanger sequencing was completed at the John Curtin School of Medicine, Australian National University, Canberra, Australia. HTS libraries were prepared as per the Illumina protocol (# 15044223 Rev. B) with modifications as previously described.^14^ HTS was performed using an Illumina MiSeq at the CSIRO Black Mountain Laboratory.

### 2.4 Data analysis

Whitefly mortality data from the bifenthrin bioassays were corrected for control mortality (0.7%–5.4%)^41^ and analysed using probit regression in Genstat 19.^42^ From this analysis, the dose‐dependent mortality response including the slope, median lethal concentration (LC_50_) estimate and associated 95% fiducial limits were determined. During the analysis, heterogeneity was checked using a chi‐square test and, if significant at the 5% level, the variance of the estimated parameter was scaled by the corresponding heterogeneity factor equal to the residual mean deviance.^43^ For each field population, their resistance ratio and associated 95% confidence interval (CI), were calculated as outlined in Robertson and Preisler.^44^

All amplicon sequencing analysis was completed using CLC Genomics Workbench v21.0 (https://digitalinsights.qiagen.com/). FastQ files were imported as joint‐paired‐end reads and quality trimmed with 0.05 quality scores (*Q* = 13). Trimmed mtCOI reads were mapped to the updated *B. tabaci* mtCOI database.^13^ Assembled contigs of the mtCOI, *ace1* and *vgsc* genes were verified using tblastn^45^ to confirm that the correct gene regions were amplified. The *vgsc* and *ace1* reads were mapped to the reference MEAM1 *vgsc* (GenBank: DQ205205.1)^25^ and *ace1* (GenBank: LC199301.1, unpublished) sequences. After mapping the amplicon reads to the *ace1* and *vgsc* reference sequences, single‐nucleotide polymorphisms (SNPs) were called at base 61 for L925I (in *vgsc* amplicon) and at bases 222, 327 and 335/336 for F331W (in *ace1* amplicon) when > 1% (variants observed at less than this frequency will not be called). Ploidy (the maximum number of different alleles expected) was set to two before a SNP was called as being present or absence in a population.

### 2.5 Phylogenetic inference

We aligned the candidate AUS I and MEAM1 partial sequences against randomly selected representatives of *B. tabaci* cryptic species partial mtCOI sequences from the updated *B. tabaci* database of Kunz *et al*.^13^ To provide confidence of our molecular species identification, we selected representatives of the Asian species including the Indonesian species (HQ457045)^13^ because of their geographic proximities to Australia, as well as representative species from the invasive clade (Indian Ocean, MEAM1, MED species complex).^9^, ^10^ Alignment was carried out using MAFFT^46^, ^47^ with default options (algorithm = auto; scoring matrix = 200PAM/*K* = 2; gap open penalty = 1.53; offset value = 0.123) within Geneious v11.1.5 and trimmed to 482 bp to match our sequence length. Trimmed and aligned sequences were exported as FASTA file for phylogenetic inference using IQTree^48^ selecting the ‘auto’ option for optimal base substitution model and the Ultrafast Bootstrap (UFBoot) option^49^ with 1000 replications for branch support. Visualisation and manipulation of phylogeny was carried out using Dendroscope 3.^50^

## 3 RESULTS

### 3.1 Species identification in *B. tabaci* complex in Australia

**FIGURE 2:**
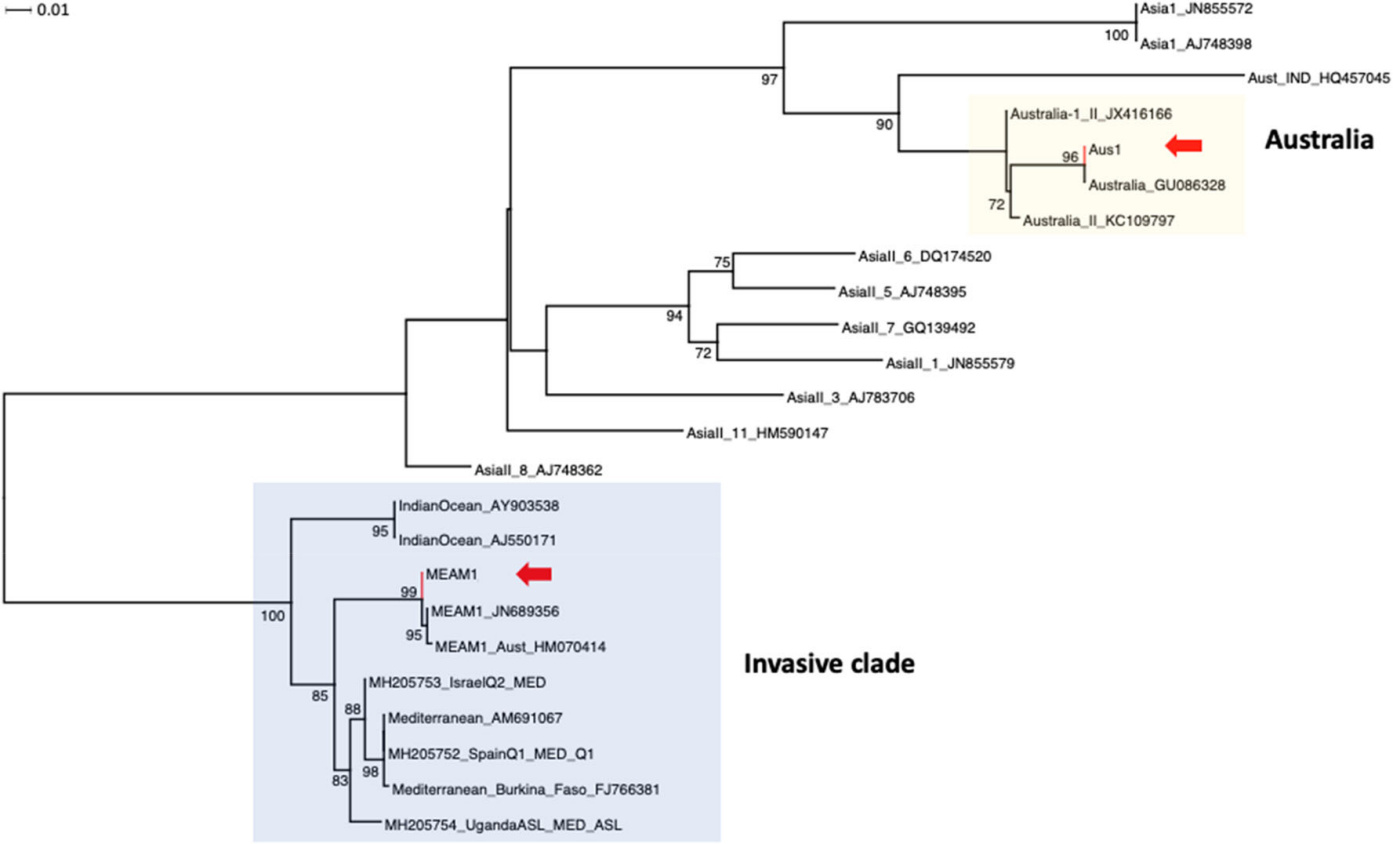
An unrooted maximum‐likelihood (ML) phylogenetic tree based on 483 bp of *Bemisia tabaci* partial mt*COI* gene sequences using IQTree.^48^ Arrows show the phylogenetic placements (with high node support values) of the two cryptic species (AUS I, MEAM1) detected in this study against selected partial mtCOI gene sequences^13^ of species including the other endemic AUS II species (JX416166, KC109797), an Indonesian species (HQ457045), various Asian species, the Indian Ocean, Mediterranean (MED) species complex and the MEAM1 species.^9^ Node confidence estimates are based on 1000 UltraFast bootstrap replications; bootstrap support > 70% are shown. Note that the AN12‐1 species sequence is 100% identical to another reported AUS I sequence (GU086328); the characterised MEAM1 species sequence is 100% identical to other MEAM1 sequences reported from countries including the USA (GU086340, HM070411), Taiwan (GU086342), Egypt (DQ133373), Japan (AB204577) and Brazil (JN689356).

Both Sanger and metabarcoding sequencing approaches were used to sequence the mtCOI region. The mtCOI contigs were confidently (sequence identity = 100%) mapped to various reported MEAM1 partial mtCOI sequences including from the USA (GU086340, HM070411), Taiwan (GU086342), Egypt (DQ133373), Japan (AB204577) and Brazil (JN689356), while the AUS I contigs shared 100% nucleotide identity with another reported AUS I sequence (GU086328). Phylogenetic analysis [best‐fit model according to BIC (5083.8948): TIMM +F + G4] further validated the candidate *B. tabaci* species mtCOI sequences as belonging to MEAM1 and AUS I (Figure 2, red arrows) with 99% and 95% bootstrap values, respectively (Figure 2). Note that the lower confidence (< 70%) for the Australia endemic species clade was due to the short sequence length used in this study.

In total, 140 whitefly field populations and 4 laboratory reference strains including ‘GU10‐1R’, ‘SU07‐1’, native ‘AN12‐1’ and ‘AY09‐1R’ were included in this study. The field populations were collected in 11 Australian cotton‐growing valleys and 4 horticultural regions between 2013 and 2021 (Table 1 and Figure 1) and sequenced to ascertain the species composition within a given population. Of the 144 populations, 142 populations contained only the invasive species MEAM1, whereas 2 populations, collected from Goondiwindi in 2016 and 2017, were mixed populations that contained AUS I and MEAM1 (with 19.6% and 7.9% AUS I individuals, respectively).

### 3.2 Frequencies of *ace1* mutations in Australian MEAM1 and AUS I populations

**FIGURE 3:**
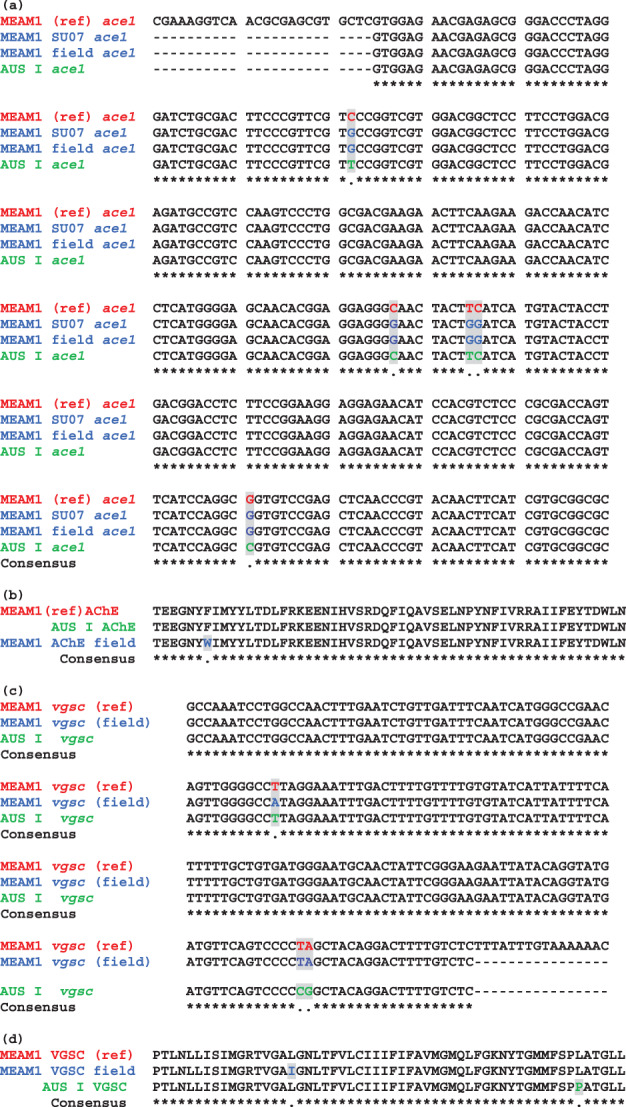
Alignment of partial *ace1* and *vgsc* nucleotide and protein sequences from *Bemisia tabaci* MEAM1 and AUS I. (a) Nucleotide *ace1* sequences from MEAM1 (LC199301.1), SU07 (susceptible laboratory population), field collected and AUS I; (b) amino acid sequences translated from the MEAM1 reference sequence (LC199301.1), AUS I and MEAM1 field populations; (c) nucleotide partial *vgsc* sequences from MEAM1 *vgsc* reference sequence (DQ205205.1), MEAM1 field populations and AUS I population; and (d) amino acid sequences translated from a susceptible MEAM1 reference sequence (DQ205205.1), MEAM1 field populations and AUS I population.

*B. tabaci* MEAM1 field populations collected from 2013 to 2021 and AN12‐1 were partially sequenced to determine the frequency of OP resistance alleles. Three nucleotide substitutions, GTC → GTG, GGC → GGG and TTC → TGG (Figure 3), were present at very high frequencies (> 99%) in all populations. The SNPs GTC → GTG and GGC → GGG are synonymous, whereas TTC → TGG results in an amino acid change from phenylalanine to tryptophan (F331W) in the MEAM1 AChE protein. The laboratory susceptible reference population ‘SU07‐1’ also showed > 99% frequency of the F331W mutation. The results indicate fixation of the F331W variant in the sampled whitefly field populations and suggest widespread OP‐resistant MEAM1 populations in NSW and Qld.

Interestingly, two Goondiwindi populations (‘Goondiwindi 17B’ and ‘Goondiwindi 16C’) did not show > 99% of the F331W variant. The SNP frequencies in these two populations were 95% for GTC → GTG, 95% for GGC → GGG and 94% for TTC → TGG (F331W) for ‘Goondiwindi 16C’ and 96% for GTC → GTG, 96% for GGC → GGG and 96% for TTC → TGG (F331W) for ‘Goondiwindi 17B’. The < 99% frequency results from these two populations are likely due to the presence of *B. tabaci* AUS I (detected by mtCOI sequencing as described above).

To support the notion that the *B. tabaci* AUS I population ‘AN12‐1’ does not possess the F331W mutation, *ace1* amplicons from the *B. tabaci* ‘AN12‐1’ population were sequenced from a pool of 60 individuals, and all were negative for the F331W mutation (Figure 3).

### 3.3 Frequencies of *vgsc* mutations in Australian MEAM1 and AUS I populations

Whitefly field populations collected from 2013 to 2021 were sequenced for pyrethroid resistance alleles. In the pyrethroid‐resistant strain ‘GU10‐1R’, we detected a 98% frequency for the non‐synonymous SNP TTA → ATA; the resulting amino acid change from leucine to isoleucine (L925I) amino acid change is associated with pyrethroid resistance.^26^ By contrast, we did not find the L925I variant in the ‘SU07‐1’ laboratory strain (the frequency for this variant is below the cut‐off of 1%).

Sequencing field populations to detect the L925I variation revealed that 43 MEAM1 populations show low frequencies between 1.0% and 7.0%, two populations (Theodore 18A and AY09‐1R) show frequencies of 27.7% and 42.1%, respectively, and three populations (Gumlu 19A, Bowen 19A and Ayr 16A) showed high frequencies of 95.1%, 95.0% and 97.5% (Table 3). The remaining field populations (*n* = 97) had no detectable resistance genes (levels of < 1%), indicating that the majority if not all MEAM1 individuals in these populations are homozygous for the leucine codon at position 925. Other known variations associated with pyrethroid resistance in *B. tabaci* MEAM1 and MED are M918V and T929V,^25^, ^26^, ^51^ but neither M918V (found in MEAM1) nor T929V (found in MED) were found in any of the populations that were sampled in our study.

**TABLE 3:** Frequency of L925I and dose–response data for bifenthrin‐resistant *Bemisia tabaci* MEAM1 field populations (collected between 2013 and 2021)

Population (generation)	Date of collection	*n* ^a^	χ^2^ (df)^b^	Slope (SE)^c^	LC_50_ (mg L^−1^)^d^	FL 95%^e^	RRs	95% CI^f^	Mortality (%) at 300 mg L^−1^	L925I frequency (%)
SU07‐1^g^	Oct 07	471	40.9 (27)	2.50 (0.30)	3.0	2.4–3.7	–	–	100	0.1
GU10‐1R^h^	Nov 10	365	34.1 (18)	3.1 (0.5)	20 000	17 747–26 292	7090	5444–9235	1	95.6
AY09‐1R	Sept 09	458	52.6 (23)	0.69 (0.1)	60.0	30.1–123.2	20.1	10.1–39.7	76.7	42.1
Ayr 16A	Jul 16	–	–	–	–	–	–	–	–	97.5
Theodore 18A	Jan 18	–	–	–	–	–	–	–	–	27.7
Gumlu 19A (G_2_)	Oct 19	303	–	–	>1000	–	>300	–	1.8	95.1
Bowen 19A (G_2_)	Oct 19	518	–	–	>1000	–	>300	–	0.9	95.0
Emerald 13B (G2)	Feb 13	308	62.9 (18)	1.57 (0.3)	20.6	9.8–33.4	6.9	4.0–11.9	97.4	0.2
St George 13A (G2)	Feb 13	303	56.2 (18)	1.39 (0.3)	32.7	17.0–54.0	10.9	6.4–18.6	93.2	0.2
St George 13B (G2)	Feb 13	269	22.8* (18)	1.49 (0.2)	21.5	14.9–28.6	7.2	4.9–10.5	98.6	0.3
St George 13C (G2)	Feb 13	276	16.1* (18)	1.60 (0.2)	21.8	16.0–28.1	7.3	5.2–10.3	97.0	0.1
Narrabri 13B (G_1_)^i^	Mar 13	290	32.4 (18)	2.30 (0.38)	15.7	10.6–21	5.3	4.4–9.2	100	2.0
St George 15C (G_1_)	Feb 15	484	40.0 (23)	1.45 (0.22)	10.8	5.3–16.8	3.6	2.1–6.3	99.0	0.2
Moree 15B (G_2_)	Apr 15	407	25.1* (23)	0.99 (0.1)	6.5	3.7–10.0	2.2	1.3–3.7	95.2	0.2
Narrabri 15A (G_2_)	Apr 15	443	37.6 (23)	1.05 (0.13)	5.5	2.6–9.3	1.8	1.0–3.4	93.0	0.3
Goondiwindi 16A (G_2_)	Mar 16	464	20.9* (23)	1.37 (0.12)	16.6	11.9–22.7	5.6	3.8–8.2	98.6	0.2
Narrabri 16B (G_2_)	Mar 16	276	21.7* (18)	1.62 (0.16)	9.4	6.8–12.9	3.2	2.2–4.6	100	1.6
Hillston 16A (G_3_)	Mar 16	424	35.5 (23)	1.72 (0.19)	4.8	3.4–6.7	1.6	1.1–2.3	98.9	0.1
Theodore 17A (G_4_)^i^	Feb 17	399	31.8 (13)	2.11 (0.29)	3.8	2.6–5.5	1.3	0.9–1.9	100	7.0
Goondiwindi 17A (G_3_)^i^	Mar 17	754	55.0 (23)	1.42 (0.13)	5.3	3.7–7.3	1.8	1.2–2.6	99.0	1.4
Goondiwindi 17B	Mar 17	–	–	–	–	–	–	–	–	2.7
Goondiwindi 17C	Mar 17	–	–	–	–	–	–	–	–	4.3
Moree 17A	Mar 17	–	–	–	–	–	–	–	–	4.0
Emerald 18A (G_3_)	Jan 18	378	21.6* (23)	1.18 (0.09)	7.8	5.6–10.7	2.6	1.8–3.9	98.9	0.5
Goondiwindi 18A	Mar 18	416	35.8* (23)	1.65 (0.18)	12.0	8.1–17.4	4.0	2.7–6.1	98.8	1.3
Dubbo 18A (G_6_)	Feb 18	366	19.3* (18)	1.39 (0.12)	5.6	4.1–7.6	1.9	1.3–2.7	100	2.1
Emerald 19B	Jan 19	–	–	–	–	–	–	–	–	2.5
Dalby 19A (G_2_)	Mar 19	242	26.9* (18)	1.16 (0.14)	3.4	2.0–5.3	1.1	0.7–1.9	100	1.3
St George 19A	Mar 19	–	–	–	–	–	–	–	–	1.0
St George 19B	Mar 19	–	–	–	–	–	–	–	–	1.2
St George 19C (G_2_)	Mar 19	505	18.0* (23)	1.37 (0.12)	4.6	3.3–6.2	1.5	1.1–2.2	98.9	0.9
Mungindi 19A (G_1_)	Mar 19	401	35.9 (23)	1.12 (0.13)	3.7	2.0–6.2	1.3	0.7–2.2	97.5	1.2
Moree 19A	Mar 19	–	–	–	–	–	–	–	–	1.4
Narrabri 19A	Mar 19	–	–	–	–	–	–	–	–	1.2
Narrabri 19C (G_1_)	Mar 19	458	36.7 (23)	1.58 (0.20)	3.3	2.1–4.9	1.1	0.7–1.7	98.9	1.0
Griffith 19A	Mar 19	466	29.9* (18)	2.13 (0.2)	8.5	6.7–10.9	2.9	2.1–3.9	100	1.3
Dubbo 19A (G_3_)	Apr 19	350	22.7* (18)	1.71 (0.16)	6.4	4.8–8.4	2.1	1.5–3.0	100	1.1
Emerald 20A (G_1_)	Dec 19	468	72.6 (23)	0.93 (0.13)	9.3	3.9–18.2	3.1	1.5–6.5	93.7	1.7
Theodore 20A (G_2_)	Jan 20	662	57.8 (23)	1.3 (0.13)	9.9	6.3–19.9	3.3	2.1–5.2	97.5	2.0
St George 20A	Mar 20	–	–	–	–	–	–	–	–	1.1
St George 20C (G_2_)	Mar 20	295	36.4 (23)	1.21 (0.16)	5.3	2.9–8.8	1.8	1.0–3.1	98.1	1.1
Goondiwindi 20A	Mar 20	–	–	–	–	–	–	–	–	1.1
Goondiwindi 20C (G_2_)	Mar 20	404	54.8 (23)	1.13 (0.16)	5.1	2.4–9.3	1.7	0.9–3.3	93.1	1.8
Moree 20C (G_2_)	Mar 20	510	38.4 (23)	0.96 (0.11)	3.8	1.9–6.5	1.3	0.7–2.3	98.1	0.8
Narrabri 20A	Mar 20	–	–	–	–	–	–	–	–	1.0
Narrabri 20B (G_2_)	Mar 20	433	30.3* (23)	0.99 (0.08)	9.9	6.5–14.3	3.3	2.1–5.2	94.2	0.9
Narrabri 20C	Mar 20	–	–	–	–	–	–	–	–	1.4
Dubbo 20A (G_4_)	Mar 20	557	55.8 (23)	1.46 (0.18)	5.3	3.2–8.2	1.8	1.1–2.9	98.5	0.9
Dalby 20A (G_3_)	Apr 20	595	63.7 (23)	0.98 (0.12)	4.8	2.3–8.4	1.6	0.8–3.0	95.0	2.5
Hillston 20A (G_5_)	Apr 20	272	53.8 (23)	1.34 (0.21)	7.9	3.9–14.9	2.7	1.4–5.1	96.3	3.7
Emerald 21A (G_2_)	Dec 20	400	47.5 (23)	0.76 (0.10)	5.2	2.1–10.6	1.8	0.6–5.4	88.5	4.7
Theodore 21A (G_2_)	Jan 21	363	24.4 (18)	2.07 (0.21)	3.8	2.9–4.8	1.3	0.9–1.7	100	1.2
St George 21A	Mar 21	–	–	–	–	–	–	–	–	1.2
St George 21B (G_4_)	Mar 21	466	39.0 (23)	1.02 (0.10)	8.8	5.1–14.1	3.0	1.8–5.0	89.7	1.1
St George 21C	Mar 21	–	–	–	–	–	–	–	–	1.3
Goondiwindi 21B (G_4_)	Mar 21	434	62.1 (23)	0.85 (0.12)	10.7	4.5–21.1	3.6	1.7–7.5	86.2	1.2
Goondiwindi 21C	Mar 21	–	–	–	–	–	–	–	–	1.0
Moree 21C (G_4_)	Mar 21	449	77.8 (23)	1.1 (0.18)	3.8	1.5–7.6	1.3	0.6–2.7	97.5	1.4
Moree 21A	Mar 21	–	–	–	–	–	–	–	–	1.2
Moree 21B	Mar 21	–	–	–	–	–	–	–	–	1.4
Narrabri 21B	Mar 21	–	–	–	–	–	–	–	–	1.2
Narrabri 21C (G_3_)	Mar 21	390	44.3 (22)	0.96 (0.12)	5.2	2.6–9.2	1.8	0.9–3.3	96.2	1.1
Hillston 21A (G_4_)	Mar 21	442	73.1 (23)	1.1 (0.16)	6.6	2.8–12.9	2.2	1.1–4.6	96.6	1.4
Griffith 21A (G_4_)	Mar 21	416	49.9 (23)	1.00 (0.13)	6.8	3.3–12.1	2.3	1.2–4.2	93.1	1.0

^a^ Number of individuals tested in the dose–response bioassay.

^b^ Chi‐square test of independence with degrees of freedom in parentheses.

^c^ Regression line of dose (mg L^−1^) against mortality.

^d^ Lethal concentration that kills 50% of the tested individuals.

^e^ 95% fiducial limit of LC_50_ value.

^f^ 95% confidence interval for RRs.

^g^ A susceptible laboratory reference population. The name represents the locality, year of collection and the order of population collected.

^h^ A population collected from Gumlu and selected with pyrethroids to create a pyrethroid‐resistant population.

^i^ Populations previously published by Hopkinson *et al*.^29^

* Statistically significant (*p* < 0.05).

The alignment of sodium channel protein sequences from MEAM1 and AUS I populations revealed a single amino acid change from leucine (in MEAM1) to proline (in AUS I). This change has not been previously documented and is likely due to the diversity of the different *B. tabaci* species (Figure 3).

### 3.4 Pyrethroid bioassay data

**FIGURE 4:**
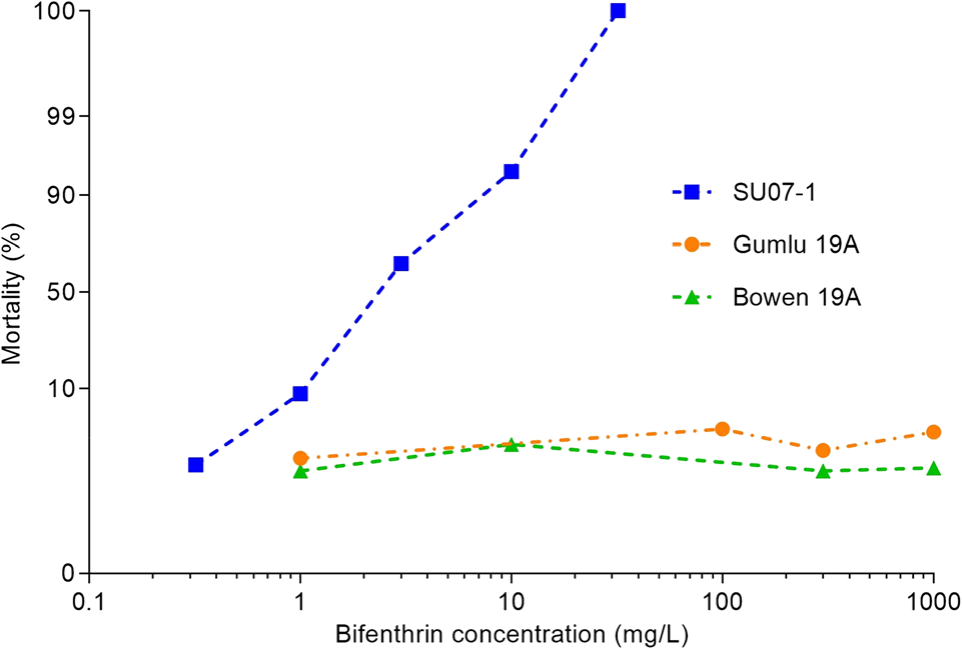
The dose–response to bifenthrin as measured by mortality for the susceptible laboratory population (SU07‐1) and two field‐collected populations (Gumlu 19A and Bowen 19A) of *Bemisia tabaci* MEAM1.

Since 2013, a total of 85 field populations have been tested using insecticide bioassays. Survivors suggesting resistance to bifenthrin were detected in 34 populations at the discriminating dose of 300 mg L^−1^ bifenthrin (Table 3). In the cotton‐growing regions under investigation, the resistance frequency is relatively low with a 86.2%–99% mortality rate at the discriminating dose (with resistance ratios between 1.1 and 5.6). By contrast, two populations that were collected in 2019 from horticultural regions (‘Gumlu 19A’ and ‘Bowen 19A’) showed high resistance (1.8% and 0.9% mortality at the discriminating dose, respectively). With these two populations, however, it was not possible to use probit analysis to estimate their respective LC_50_ values, associated 95% fiducial limits or slopes, because there was no increase in mortality in response to dose (Figure 4). At locations with high allele frequencies (for example, near Gumlu and Bowen) there was good agreement between the allele frequencies and bioassay results. However, because we sampled populations with an intermediate resistance allele frequency and bioassay survival, a meaningful statistical correlation was not observed.

## 4 DISCUSSION

Here, we provide a comprehensive overview of pyrethroid and OP resistance levels in Australian *B. tabaci* field populations, sampled between 2013 and 2021. Our study combines molecular and bioassay approaches to better characterise species composition and insecticide resistance status. We show that the *B. tabaci* MEAM1 species is common in cotton fields across the eastern states of Australia. By contrast, the endemic *B. tabaci* species AUS I was detected only rarely. Resistance to insecticides was analysed via HTS of amplicons and, for selected populations, confirmed using laboratory‐based bioassays. Our findings show that HTS and mtCOI molecular diagnostic markers can be used reliably for identifying whitefly cryptic species and analysing mixed *Bemisia* populations, in our case, mixed field populations containing MEAM1 and AUS I. However, to accurately identify the species composition in a mixed field population, HTS reads are warranted.

### 4.1 Species status

We found that MEAM1 was the only invasive species present in field samples collected between 2013 and 2021. The endemic species AUS I was very rarely found, and if AUS I was present, it was always found in mixed populations dominated by MEAM1. The finding confirms that AUS I has been largely displaced along the east coast of Australia by MEAM1.^52^ However, our findings demonstrate that AUS I is still present in the Goondiwindi region, a finding that is in line with recent reports from other cotton‐production regions.^29^, ^32^ The survival of AUS I in regions where MEAM1 is now the dominant whitefly on cotton may be linked to differences in host plant use,^32^ or to its inferior reproductive performance compared with MEAM1.^32^ Further, AUS I is suspected to be more susceptible to insecticides than MEAM1. Our surveys were timed to collect whiteflies for insecticide resistance (we usually collected after the insecticide sprays), which likely biases the collection towards MEAM1.

Apart from MEAM1, we did not detect other invasive *B. tabaci* species despite a previous report suggesting the presence of the Asia II species on bellvine (*Ipomoea plebcia*) near Emerald, Qld.^32^ Although the *B. tabaci* species complex as a whole is regarded as highly polyphagous, recent studies showed that individual whitefly species may have specific host plant preferences.^32^, ^53^ Although various *B. tabaci* cryptic species within the Asia II clade have been reported from cotton elsewhere, we did not detect it in our study.^54^, ^55^

In line with other whitefly surveillance studies,^29^, ^56^ we did not detect MED, but it should be noted (as mentioned above) that our study largely focused on sampling cotton fields. Furthermore, MED has been detected in New Zealand^9^ and MED/Asia II are endemic in several Southeast Asian countries.^57^, ^58^ An invasion of MED could be difficult to control because the species can be more resistant than MEAM1 to pyriproxyfen and imidacloprid,^59^, ^60^ insecticides currently used to control MEAM1 in Australia. Continued on‐farm surveillance (in cotton fields and other fields) along with accurate pre‐border species identification is vital to protect the Australian cotton industry.

### 4.2 Organophosphate resistance

The F331W variant associated with OP resistance has also been found in resistant MEAM1 populations from Israel^17^ and in resistant MED populations from Crete.^16^ Our results show that F331W is present in all sampled Australian MEAM1 individuals, indicating a fixation of this resistance allele. There are two synonymous SNPs, not been reported previously, that accompanied the non‐synonymous SNP (F331W) and appeared at > 99% frequencies in all samples. These two synonymous SNPs are unlikely to contribute to the resistance against OP but are potentially useful markers to differentiate between Australian and other MEAM1 populations.

Our results, along with previous findings,^61^ support the hypothesis that MEAM1 arrived in Australia with an OP resistance allele, probably already at fixation. Invasions of OP‐resistant MEAM1 populations have also been reported from elsewhere in the world^62^ and in invasive populations of MED.^63^, ^64^ OP resistance in AUS I has never been documented, and it is possible that AUS I was displaced by MEAM1 before resistance against OPs could evolve. However, further studies are required to verify this hypothesis.

### 4.3 Pyrethroid resistance

In this study, the *vgsc* mutation L925I is linked to pyrethroid resistance and was found in several field populations. It was detected in all cotton‐production regions surveyed, but both bioassay and molecular evidence indicate the frequency of resistance is low. This finding could be linked to a reduction in the use of broad‐spectrum insecticides, including pyrethroids, that followed the adoption of transgenic cotton, especially Bollgard II in 2004–2005,^65^ which predates the emergence of MEAM1 as a major pest across all Australian cotton‐production valleys.^65^

By contrast, the most recent populations that collected from horticulture operations in North Qld, ‘Gumlu 19A’ and ‘Bowen 19A’, had > 95% frequencies for the resistance marker L925I, indicating widespread resistance to pyrethroids. However, such high levels of pyrethroid resistance were not detected in populations from Gatton or Griffith (also regions with significant areas of horticultural production). Thus, additional sampling is required before we can describe the spatial distribution of pyrethroid resistance in horticulture.

Taken as a whole, our bioassay results confirm findings obtained through sequencing, for example the frequency of the L925I allele suggests that a pyrethroid‐resistant phenotype is widespread but not dominant; only from the intensive horticultural region surrounding Bowen were highly resistant populations detected. Unfortunately, it was not possible to perform a comprehensive statistical analysis to compare the bioassay and sequencing approaches because our survey found very few samples with intermediate frequencies. It is, however, worth noting that in the few populations where resistance was high, there was a good agreement between the two approaches.

This study demonstrates that the discriminating dose developed in our earlier study^29^ is effective at detecting resistance phenotypes and distinguishing them from populations that are largely comprised of susceptible individuals. The profile of the bioassay results combined with the presence of known resistance alleles suggests a target site mechanism. However, alternative resistance mechanisms such as metabolic resistance may be present, as there is evidence that esterase‐based detoxification can play a role in pyrethroid resistance (Permethrin) in MEAM1.^18^

It seems that, at least for now, dose–response bioassays remain core to the identification of resistance in field populations, but molecular approaches can deliver rapid assessments of large numbers of samples and do not require live insect bioassays. Phenotypic bioassays enable the measurement of resistance levels irrespective of the mechanism; however, knowledge of the baseline susceptibility of natural field populations is crucial. This may not exist and the bioassay approach can be time and labour intensive. Molecular approaches offer a rapid, high‐throughput complement to bioassays especially in situations where a common well‐characterised resistance mechanism is known. Furthermore, through mass scanning of known resistance alleles, we show that early detection of potential phenotypic resistance in field samples is possible, and that the metabarcoding approach is especially well‐suited for small and otherwise difficult to identify insect species such as whiteflies.

## CONFLICT OF INTEREST

The authors declared no conflict of interest.

## Supporting information


**Table S1**. Metadata associated with whitefly collections and analysis results.

## Data Availability

The data that support the findings of this study are available from the corresponding author upon reasonable request.
