# Supplementary material for: Screening for insecticide resistance in Australian field populations of Bemisia tabaci (Hemiptera: Aleyrodidae) using bioassays and DNA sequencing
Source: Pest Manag Sci. 2022 Jun 3;78(8):3248–59. doi: 10.1002/ps.6906 (PMC9546000; doi:10.1002/ps.6906)
Supplement: Supplementary file 1 — Table S1. Metadata associated with whitefly collections and analysis results. [file PS-78-3248-s001.docx]

| Collection | | | | | Bioassay | | | | | Molecular | | | | |
| --- | --- | --- | --- | --- | --- | --- | --- | --- | --- | --- | --- | --- | --- | --- |
| Population | Date | Lat. | Long. | host plant | Date | Gen. | CM (%) | LC_50_ (mg/L) | Mortality (%) - 300 (mg/L) | Date preserved | Gen. preserved | *n* | Method | L925I frequency (%) |
| SU07-1 | 8/10/2007 | -35.4163° | 149.0668° | poinsettia | 23/03/2010 | G_25_# | 1 | 3.5 | 100 |  |  |  |  |  |
|  |  |  |  |  | 28/01/2014 | G_55_ | 0 | 3 | 100 |  |  |  |  |  |
|  |  |  |  |  | 12/06/2018 | G_108_ | 0 | 2.5 | 100 | 10/10/18 | G_111_# | 20 | NGS‡ | 0.11 |
|  |  |  |  |  | 17/11/2020 | G_135_ | 0 | 2.6 | 100 |  |  |  |  |  |
| AY09-1R | 28/09/2009 | -19.6233° | 147.3461° | melon | 20/06/2018 | G_93_ | 2.1 | 60 | 76.7 | 10/10/18 | G_97_ | 20 | NGS | 42.1 |
| GU10-1R* | 10/11/2010 | -19.8839° | 147.6895° | melon | 27/10/2011 | G_9_ | 0.9 | 21121 | 1 | 10/10/18 | G_84_ | 20 | NGS | 95.5 |
| AN12-1 | 4/06/2012 | -24.8587° | 152.3481° | painted spurge | not tested | | | | | 11/09/2012 | G_3_ | 20 | NGS and Sanger | 0.14 |
| St George13A | 13/02/2013 | -28.1021° | 148.7167° | cotton | 22/04/2013 | G_2_ | 0 | 32.7 | 93.2 | 18/07/2013 | G_5_ | 20 | NGS | 0.22 |
| St George13B | 13/02/2013 | -28.0847° | 148.7132° | cotton | 22/04/2013 | G_2_ | 1.4 | 21.5 | 98.6 | 18/07/2013 | G_5_ | 20 | NGS | 0.3 |
| St George13C | 13/02/2013 | -28.0952° | 148.6826° | cotton | 22/04/2013 | G_2_ | 0 | 21.8 | 97 | 18/07/2013 | G_5_ | 20 | NGS | 0.13 |
| Emerald13A | 19/02/2013 | -23.5127° | 148.5226° | cotton | 6/05/2013 | G_2_ | 0 | 31.8 | 100 | 22/07/2013 | G_5_ | 20 | NGS | 0.18 |
| Emerald13B | 19/02/2013 | -23.5129° | 148.0864° | cotton | 6/05/2013 | G2 | 0 | 20.6 | 97.4 | 22/07/2013 | G_5_ | 20 | NGS | 0.15 |
| Theodore13A | 20/02/2013 | -24.9549° | 150.0801° | cotton | 6/05/2013 | G_2_ | 0 | 29.2 | 100 | 22/07/2013 | G_5_ | 20 | NGS | 0.13 |
| Narrabri13A | 11/03/2013 | -30.2102° | 149.3990° | cotton | 8/04/2013 | G_1_ | 2.3 | 6.3 | 100 | 24/07/2013 | G_5_ | 20 | NGS | 0.38 |
| Narrabri13B | 11/03/2013 | -30.2776° | 149.4868° | bladder ketmia | 8/04/2013 | G_1_ | 0 | 15.7 | 100 | 24/07/2013 | G_5_ | 20 | NGS | 1.95 |
| Moree13A | 12/03/2013 | -29.4020° | 149.9521° | cotton | 17/04/2013 | G_1_ | 0 | 7.4 | 100 | 29/07/2013 | G_5_ | 20 | NGS | 0.28 |
| Moree13B | 12/03/2013 | -29.4184° | 149.8682° | cotton | 17/04/2013 | G_1_ | 0 | 11.6 | 100 | 29/07/2013 | G_5_ | 20 | NGS | 0.1 |
| Gatton13A | 17/04/2013 | -27.5446° | 152.3289° | tomato | not tested | | | | | 24/07/2013 | G_4_ | 20 | NGS | 0.1 |
| Emerald15A | 22/01/2015 | -23.5068° | 148.0974° | cotton | 13/05/2015 | G_3_ | 0.9 | 12.92 | 100 | 27/11/2015 | G_11_ | 1 | Sanger | Sanger |
| Emerald15B | 22/01/2015 | -23.5456° | 148.0945° | cotton | 13/05/2015 | G_3_ | 0 | 16.11 | 100 | 27/11/2015 | G_11_ | 1 | Sanger | Sanger |
| St George15A | 24/02/2015 | -28.2644° | 148.5173° | cotton | 25/03/2015 | G_1_ | 0 | 12.3 | 100 | 8/12/2015 | G_9_ | 20 | NGS | 0.17 |
| St George15B | 24/02/2015 | -28.2334° | 148.5704° | cotton | 25/03/2015 | G_1_ | 2.8 | 13.6 | 100 | 8/12/2015 | G_9_ | 20 | NGS | 0.2 |
| St George15C | 24/02/2015 | -28.1116° | 148.6467° | cotton | 25/03/2015 | G_1_ | 1 | 10.8 | 99 | 8/12/2015 | G_9_ | 20 | NGS | 0.17 |
| Theodore15A | 3/03/2015 | -24.9550° | 150.0803° | cotton | 30/03/2015 | G_1_ | 1.1 | 5 | 100 | 26/11/2015 | G_9_ | 20 | NGS | 0.16 |
| Griffith15A | 26/03/2015 | -34.1198° | 145.9863° | melon | 29/04/2015 | G_1_ | 0.9 | 15.1 | 100 | 8/12/2015 | G_9_ | 40 | NGS | 0.23 |
| Dalby15A | 1/04/2015 | not recorded | | cotton | 15/06/2015 | G_2_ | 2.2 | 7.2 | 100 | 4/12/2015 | G_8_ | 10 | NGS | 0.21 |
| Narrabri15A | 2/04/2015 | -30.2097° | 149.4070° | cotton | 17/06/2015 | G_2_ | 0 | 5.4 | 93 | 26/11/2015 | G_8_ | 20 | NGS | 0.31 |
| Narrabri 15B | 2/04/2015 | -30.1590° | 149.6216° | cotton | 17/06/2015 | G_2_ | 3.9 | 4.9 | 100 | 26/11/2015 | G_8_ | 20 | NGS | 0.18 |
| Moree15A | 2/04/2015 | -29.3426° | 149.7176° | cotton | 9/06/2015 | G_2_ | 0 | 7.4 | 100 | 23/11/2015 | G_8_ | 20 | NGS | 0.17 |
| Moree15B | 2/04/2015 | -29.3668° | 149.8360° | cotton | 9/06/2015 | G_2_ | 3.7 | 6.5 | 95.2 | 23/11/2015 | G_8_ | 20 | NGS | 0.2 |
| Goondiwindi15A | 9/04/2015 | -28.7142° | 150.4464° | cotton | 24/06/2015 | G_2_ | 1 | 6.8 | 100 | 4/12/2015 | G_8_ | 10 | NGS | 0.21 |
| Goondiwindi15B | 9/04/2015 | -28.6330° | 150.3399° | cotton | 24/06/2015 | G_2_ | 0 | 3.7 | 100 | 4/12/2015 | G_8_ | 20 | NGS | 0.16 |
| Goondiwindi15C | 9/04/2015 | -28.5918° | 150.3096° | cotton | 24/06/2015 | G_2_ | 2.2 | 4.3 | 100 | 4/12/2015 | G_8_ | 10 | NGS | 0.16 |
| St George16A | 9/02/2016 | -28.1307° | 148.6675° | mungbean | 4/05/2016 | G3 | 0 | 4 | 100 | 4/03/2016 | G_1_ | 20 | NGS | 0.2 |
| St George16B | 9/02/2016 | -28.1895° | 148.7065° | cotton | 1/06/2016 | G4 | 5.4 | 3.2 | 100 | 4/03/2016 | G_1_ | 20 | NGS | 0.21 |
| St George16C | 9/02/2016 | -28.0847° | 148.6537° | cotton | 4/05/2016 | G_3_ | 0.8 | 3.4 | 100 | 4/03/2016 | G_1_ | 1 | Sanger | Sanger |
| Moree16A | 11/02/2016 | -29.3249° | 149.6446° | cotton | 4/04/2016 | G_2_ | 1.2 | 7.5 | 100 | 8/03/2016 | G_1_ | 20 | NGS | 0.21 |
| Moree16B | 11/02/2016 | -29.3630° | 149.8195° | cotton | 4/04/2016 | G_2_ | 1.1 | 9.3 | 100 | 8/03/2016 | G_1_ | 20 | NGS | 0.13 |
| Emerald16A | 26/02/2016 | -23.5272° | 148.1896° | cotton | 19/05/2016 | G_3_ | 4.2 | 3.2 | 100 | 24/03/2016 | G_1_ | 1 | Sanger | Sanger |
| Theodore16B | 26/02/2016 | -24.9369° | 149.9905° | cotton | 19/05/2016 | G_3_ | 3 | 1.5 | 100 | 24/03/2016 | G_1_ | 1 | Sanger | Sanger |
| Goondiwindi16A | 3/03/2016 | -28.7300° | 150.4300° | cotton | 27/04/2016 | G_2_ | 2.7 | 16.6 | 98.6 | 31/03/2016 | G_1_ | 20 | NGS | 0.16 |
| Goondiwindi16B | 3/03/2016 | -28.6253° | 150.3240° | cotton | 27/04/2016 | G_2_ | 0 | 5.2 | 100 | 31/03/2016 | G_1_ | 20 | NGS | 0.27 |
| Goondwindi16C | 3/03/2016 | -28.5875° | 150.2808° | cotton | 27/04/2016 | G_2_ | 0 | 5.6 | 100 | 31/03/2016 | G_1_ | 20 | NGS | 0.23 |
| Hillston16A | 4/03/2016 | -33.4040° | 145.9238° | cotton | 3/08/2016 | G_4_ | 0 | 4.8 | 99 | 31/03/2016 | G_1_ | 10 | NGS | 0.12 |
| Narrabri16A | 10/03/2016 | -30.1562° | 149.6590° | cotton | 3/05/2016 | G_2_ | 0 | 7.7 | 100 | 6/04/2016 | G_1_ | 10 | NGS | 0.14 |
| Narrabri16B | 10/03/2016 | -30.2070° | 149.4019° | cotton | 3/05/2016 | G_2_ | 2.6 | 9.4 | 100 | 6/04/2016 | G_1_ | 10 | NGS | 1.63 |
| Goondwindi16D | 13/03/2016 | -28.7013° | 149.8217° | cotton | not tested | | | | | 13/04/2016 | G_1_ | 20 | NGS | 0.15 |
| Croppa Creek | 23/03/2016 | -29.1589° | 150.3502° | cotton | not tested | | | | | 27/04/2016 | G_2_ | 20 | NGS | 0.2 |
| Mungindi16A | 13/06/2016 | -28.6336° | 149.2688° | cotton | not tested | | | | | 13/04/2016 | G_1_ | 20 | NGS | 0.18 |
| Ayr16A | 26/07/2016 | -19.8111° | 147.2253° | melon | not tested | | | | | 15/02/2016 | G_2_ | 20 | NGS | 97.48 |
| Emerald17A | 1/02/2017 | -23.4442° | 148.1496° | cotton | 19/06/2017 | G_4_ | 0 | 4.9 | 100 | 28/07/2017 | G_6_ | 20 | NGS | 0.14 |
| Theodore17A | 2/02/2017 | -24.9138° | 150.0736° | cotton | 19/06/2017 | G_4_ | 0.7 | 3.8 | 100 | 28/07/2017 | G_4_ | 20 | NGS | 6.94 |
| Theodore17B | 2/02/2017 | -24.9361° | 149.9846° | cotton | not tested | | | | | 27/02/2017 | G_1_ | 10 | NGS | 0.1 |
| St George17A | 16/02/2017 | -28.1200° | 148.6998° | cotton | not tested | | | | | 17/02/2017 | G_0_ | 10 | NGS | 0.18 |
| St George17B | 16/02/2017 | -28.0914° | 148.7704° | cotton | 19/06/2017 | G_4_ | 2.5 | 4.8 | 100 | 17/02/2017 | G_0_ | 20 | NGS | 0.21 |
| St George17C | 16/02/2017 | -27.8331° | 148.6053° | cotton | not tested | | | | | 17/02/2017 | G_0_ | 20 | NGS | 0.16 |
| Goondiwindi17A | 3/03/2017 | -28.6115° | 150.1430° | cotton | 12/06/2017 | G_3_ | 0 | 5.3 | 99.4 | 19/07/2017 | G_4_ | 20 | NGS | 1.37 |
| Goondiwindi17B | 3/03/2017 | -28.5940° | 150.2710° | cotton | not tested | | | | | 3/03/2017 | G_0_ | 20 | NGS | 2.66 |
| Goondiwindi17C | 3/03/2017 | -28.6330° | 150.3390° | cotton | not tested | | | | | 19/07/2017 | G_4_ | 20 | NGS | 4.27 |
| Dalby17A | 9/03/2017 | -27.8164° | 151.4208° | cotton | 11/08/2017 | G_4_ | 3.3 | 3.9 | 100 | 9/03/2017 | G_0_ | 20 | NGS | 0.19 |
| Hillston17A | 17/03/2017 | -33.4040° | 145.9238° | cotton | 11/08/2017 | G_4_ | 0.9 | 6.6 | 100 | 27/03/2017 | G_1_ | 20 | NGS | 0.16 |
| Narrabri17A | 29/03/2017 | -30.1990° | 149.4040° | cotton | 5/06/2017 | G_2_ | 0 | 4 | 100 | 29/03/2017 | G_0_ | 20 | NGS | 0.16 |
| Narrabri17B | 29/03/2017 | -30.1240° | 149.5700° | cotton | not tested | | | | | 29/03/2017 | G_0_ | 20 | NGS | 0.17 |
| Moree17A | 29/03/2017 | -29.2903° | 149.7569° | cotton | not tested | | | | | 29/03/2017 | G_0_ | 20 | NGS | 0.14 |
| Moree17B | 29/03/2017 | -29.3210° | 149.7159° | cotton | 4/05/2017 | G_1_ | 3.6 | 4.3 | 100 | 29/03/2017 | G_0_ | 20 | NGS | 0.13 |
| Moree17C | 16/05/2017 | -29.3880° | 149.9098° | cotton | not tested | | | | | 14/09/2017 | G_3_ | 40 | NGS | 0.19 |
| Moree17D | 9/06/2017 | -29.5777° 149.3184° | | cotton | not tested | | | | | 31/07/2017 | G_2_ | 20 | NGS | 0.17 |
| Moree17E | 4/08/2017 | -29.3221° | 149.7291° | Common sowthistle | not tested | | | | | 14/09/2017 | G_1_ | 10 | NGS | 0.14 |
| Moree17F | 7/08/2017 | -29.1574° | 149.2151° | Common sowthistle | not tested | | | | | 14/09/2017 | G_1_ | 20 | NGS | 0.35 |
| Emerald18A | 30/01/2018 | -23.4982° | 148.0831° | cotton | 15/05/2018 | G_3_ | 0.9 | 8.8 | 98.9 | 7/02/2018 | G_0_ | 20 | NGS | 0.54 |
| Theodore18A | 31/01/2018 | -24.9506° | 150.0864° | cotton | 15/05/2018 | G_3_ | 1.9 | 1.8 | 100 | 7/02/2018 | G_0_ | 20 | NGS | 0.08 |
| Theodore18A-P32 |  |  |  |  |  |  |  |  |  | 23/05/2018 | G_3_ | 20 | NGS | 27.74 |
| Dubbo18A | 6/02/2018 | -31.8130° | 147.7255° | cotton | 17/09/2018 | G_6_ | 2 | 5.6 | 100 | 19/03/2018 | G_1_ | 20 | NGS | 2.1 |
| St George18A | 1/03/2018 | -28.1118° | 148.6881° | cotton | 15/05/2018 | G_2_ | 0 | 1.9 | 100 | 7/03/2018 | G_0_ | 20 | NGS | 0.83 |
| St George18A-P32 |  |  |  |  |  |  |  |  |  | 23/05/2018 | G_3_ | 20 | NGS | 0.04 |
| St George18A-P100 |  |  |  |  |  |  |  |  |  | 05/07/2018 | G_4_ | 20 | NGS | 0.09 |
| St George18B | 1/03/2018 | -28.1195° | 148.7127° | cotton | not tested | | | | | 7/03/2018 | G_0_ | 20 | NGS | 0.15 |
| St George18C | 1/03/2018 | -28.1722° | 148.7691° | cotton | not tested | | | | | 7/03/2018 | G_0_ | 20 | NGS | 0.06 |
| Narrabri18A | 12/03/2018 | -30.1716° | 149.6504° | cotton | 4/07/2018 | G_3_ | 2.2 | 4.9 | 100 | 23/03 2018 | G_0_ | 20 | NGS | 0.21 |
| Narrabri18B | 12/03/2018 | -30.1946° | 149.6203° | cotton | not tested | | | | | 23/03 2018 | G_0_ | 20 | NGS | 0.07 |
| Narrabri18C | 12/03/2018 | -30.1497° | 149.5091° | cotton | not tested | | | | | 23/03 2018 | G_0_ | 20 | NGS | 0.08 |
| Moree18A | 14/03/2018 | -29.4847° | 149.5012° | cotton | 4/07/2018 | G_3_ | 0 | 4.7 | 100 | 23/03 2018 | G_0_ | 20 | NGS | 0.07 |
| Moree18B | 14/03/2018 | -29.3277° | 149.7092° | cotton | not tested | | | | | 23/03 2018 | G_0_ | 20 | NGS | 0.09 |
| Moree18C | 14/03/2018 | -29.2942° | 149.8803° | cotton | not tested | | | | | 23/03 2018 | G_0_ | 20 | NGS | 0.19 |
| Goondwindi18A | 15/03/2018 | -28.6275° | 150.3318° | cotton | 4/07/2018 | G_3_ | 1.1 | 12.01 | 98.8 | 23/03/2018 | G_0_ | 20 | NGS | 1.33 |
| Goondiwindi18B | 15/03/2018 | -28.7297° | 150.4270° | cotton | not tested | | | | | 23/03/2018 | G_0_ | 20 | NGS | 0.15 |
| Griffith18A | 10/05/2018 | -34.5398° | 146.1898° | cotton | 7/09/2018 | G_3_ | 3.1 | 8.9 | 98.8 | 25/06/2018 | G_1_ | 20 | NGS | 0.26 |
| Theodore19A | 21/01/2019 | -24.9050° | 150.0791° | cotton | 26/03/2019 | G_2_ | 0 | 1.7 | 100 | 1/03/2019 | G_1_ | 20 | NGS | 0.81 |
| Emerald19A | 22/01/2019 | -23.6060° | 148.5201° | cotton | 20/02/2019 | G_1_ | 2.7 | 3.5 | 100 | 1/03/2019 | G_1_ | 20 | NGS | 0.84 |
| Emerald19B | 30/01/2019 | -23.7251° | 148.1420° | cotton | not tested | | | | | 7/02/2019 | G_0_ | 20 | NGS | 2.5 |
| Emerald19B-Surv |  |  |  |  |  |  |  |  |  | 19/09/2019 | G_2_ | 20 | NGS | 1.2 |
| Emerald19B-P |  |  |  |  |  |  |  |  |  | 4/09/2019 | G_1_ | 20 | NGS | 1.98 |
| Dalby 19A | 1/03/2019 | -27.2431° | 151.1720° | cotton | 8/05/2019 | G_2_ | 3.1 | 3.4 | 100 | 11/04/2019 | G_1_ | 20 | NGS | 1.32 |
| Mungindi19A | 1/03/2019 | -28.6631° | 149.2080° | cotton | 26/03/2019 | G_1_ | 0 | 3.7 | 97.5 | 4/03/2019 | G_0_ | 20 | NGS | 1.19 |
| St George19A | 6/03/2019 | -28.1267° | 148.6650° | cotton | not tested | | | | | 14/03/2019 | G_0_ | 20 | NGS | 1.02 |
| St George19B | 6/03/2019 | -28.1540° | 148.6970° | cotton | not tested | | | | | 14/03/2019 | G_0_ | 20 | NGS | 1.19 |
| St George19C | 6/03/2019 | -28.1661° | 148.6911° | cotton | 15/05/2019 | G_2_ | 1 | 4.6 | 98.9 | 14/03/2019 | G_0_ | 20 | NGS | 0.88 |
| Goondiwindi19A | 11/03/2019 | -28.5730° | 150.2741° | cotton | not tested | | | | | 4/09/2019 | G_5_ | 20 | NGS | 0.89 |
| Goondiwindi19B | 11/03/2019 | -28.6148° | 150.2553° | cotton | 24/04/2019 | G_1_ | 1.2 | 2.8 | 100 | 19/03/2019 | G_0_ | 20 | NGS | 0.77 |
| Goondiwindi19C | 11/03/2019 | -29.5944° | 149.9584° | cotton | not tested | | | | | 4/09/2019 | G_5_ | 20 | NGS | 0.91 |
| Goondiwindi19C-P |  |  |  |  |  |  |  |  |  | 26/09/2019 | G_2_ | 20 | NGS | 1.3 |
| Moree19A | 15/03/2019 | -29.2821° | 149.6410° | cotton | not tested | | | | | 25/03/2019 | G_0_ | 20 | NGS | 1.44 |
| Moree19B | 15/03/2019 | -29.3006° | 149.8240° | cotton | 24/04/2019 | G_1_ | 0 | 6.8 | 100 | 4/09/2019 | G_5_ | 20 | NGS | 0.97 |
| Moree19C | 15/03/2019 | -29.3930° | 149.9081° | cotton | not tested | | | | | 4/09/2019 | G5 | 20 | NGS | 0.89 |
| Narrabri19A | 21/03/2019 | -30.2011° | 149.3941° | cotton | not tested | | | | | 29/03/2019 | G_0_ | 20 | NGS | 1.21 |
| Narrabri19B | 21/03/2019 | -30.1732° | 149.4123° | cotton | not tested | | | | | 29/03/2019 | G_0_ | 20 | NGS | 0.8 |
| Narrabri19C | 21/03/2019 | -30.1880° | 149.3751° | cotton | 24/04/2019 | G_1_ | 0 | 3.3 | 98.9 | 29/03/2019 | G_0_ | 20 | NGS | 0.99 |
| Griffith19A | 1/04/2019 | not recorded | | cotton | 17/07/2019 | G_3_ | 0 | 8.5 | 100 | 18/06/2019 | G_2_ | 20 | NGS | 1.25 |
| Dubbo19A | 15/04/2019 | -31.8130° | 147.7255° | cotton | 31/07/2019 | G_3_ | 1.1 | 6.3 | 100 | 4/07/2019 | G_2_ | 20 | NGS | 1.08 |
| Bowen19A | 29/10/2019 | -20.0086° | 148.1957° | cucurbit | 29/01/2020 | G_2_ | 0 | n/a | 0.9 | 13/12/2019 | G_1_ | 20 | NGS | 95.01 |
| Bowen19A-pymetrozine | 29/10/2019 |  |  |  |  |  |  |  |  | 25/03/2020 | G_5_ | 20 | NGS | 95.15 |
| Bowen19A-imidacloprid | 29/10/2019 |  |  |  |  |  |  |  |  | 25/03/2020 | G_5_ | 20 | NGS | 96.45 |
| Gumlu19A | 30/10/2019 | -19.8222° | 147.2234° | melon | 2/12/2019 | G_1_ | 0 | n/a | 1.8 | 9/12/2019 | G_1_ | 20 | NGS | 95.07 |
| Gumlu19A-pymetrozine | 30/10/2019 |  |  |  |  |  |  |  |  | 25/03/2020 | G_5_ | 20 | NGS | 97.42 |
| Gumlu19A-imidacloprid | 30/10/2019 |  |  |  |  |  |  |  |  | 25/03/2020 | G_5_ | 20 | NGS | 97.04 |
| Emerald20A | 18/12/2019 | -23.4749° | 148.1690° | cotton | 14/01/2020 | G_1_ | 1.8 | 9.3 | 93.7 | 23/12/2019 | G_0_ | 20 | NGS | 1.68 |
| Theodore20A | 31/01/2020 | -24.9105° | 150.0778° | cotton | 25/03/2020 | G_2_ | 2.1 | 9.9 | 97.6 | 10/02/2020 | G_0_ | 20 | NGS | 1.95 |
| Goondiwindi20A | 11/03/2020 | -28.6270° | 150.2782° | cotton | not tested | | | | | 23/04/2020 | G_1_ | 20 | NGS | 1.11 |
| Goondiwindi20C | 11/03/2020 | -28.9571° | 150.3911° | cotton | 20/05/2020 | G_2_ | 0 | 5.1 | 93.1 | 23/04/2020 | G_1_ | 20 | NGS | 1.82 |
| St George20A | 16/03/2020 | -28.1330° | 148.6644° | cotton | not tested | | | | | 11/08/2020 | G_4_ | 20 | NGS | 1.07 |
| St George20B | 16/03/2020 | -28.1892° | 148.6924° | cotton | not tested | | | | | 11/08/2020 | G4 | 20 | NGS | 0.83 |
| St George20C | 16/03/2020 | -28.1061° | 148.6844° | cotton | 20/05/2020 | G_2_ | 0 | 5.3 | 98.1 | 13/10/2020 | G_6_ | 20 | NGS | 1.12 |
| Moree20A | 18/03/2020 | -29.4186° | 149.9757° | cotton | not tested | | | | | 23/04/2020 | G_1_ | 20 | NGS | 0.72 |
| Moree20C | 18/03/2020 | -29.3825° | 149.7237° | cotton | 20/05/2020 | G_2_ | 2.8 | 3.8 | 98.1 | 23/04/2020 | G_1_ | 20 | NGS | 0.81 |
| Dubbo20A | 18/03/2020 | -32.3376° | 148.0249° | cotton | 12/08/2020 | G_4_ | 0 | 5.26 | 98.4 | 19/08/2020 | G_4_ | 20 | NGS | 0.88 |
| Narrabri20A | 23/03/2020 | -30.1450° | 149.5912° | cotton | not tested | | | | | 6/10/2020 | G_5_ | 20 | NGS | 1.04 |
| Narrabri20B | 23/03/2020 | -30.2244° | 149.5572° | cotton | 27/05/2020 | G_2_ | 4.5 | 9.9 | 94.2 | 11/08/2020 | G_4_ | 20 | NGS | 0.93 |
| Narrabri20C | 24/03/2020 | -30.1372° | 149.5399° | cotton | not tested | | | | | 11/08/2020 | G_4_ | 20 | NGS | 1.36 |
| Moree20B | 24/03/2020 | -29.4988° | 150.1187° | cotton | not tested | | | | | 19/08/2020 | G_5_ | 20 | NGS | 0.84 |
| Hillston20A | 14/04/2020 | -33.4040° | 145.9238° | cotton | 7/10/2020 | G_5_ | 0 | 7.9 | 96.3 | 16/09/2020 | G_4_ | 20 | NGS | 3.66 |
| Dalby 20A | 29/04/2020 | -27.3182° | 151.2534° | cotton | 12/08/2020 | G_3_ | 1.6 | 4.8 | 95 | 14/08/2020 | G_3_ | 20 | NGS | 2.53 |
| Emerald21A | 15/12/2020 | -23.6109° | 148.5226° | cotton | 10/02/2021 | G_2_ | 0 | 5.2 | 88.5 | 1/03/2021 | G_2_ | 20 | NGS | 4.72 |
| Theodore21A | 6/01/2021 | -24.9376° | 149.9935° | cotton | 1/03/2021 | G_2_ | 0 | 3.8 | 100 | 11/02/2021 | G_1_ | 20 | NGS | 1.21 |
| St George21A | 2/03/2021 | -28.1028° | 148.7628° | cotton | not tested | | | | | 17/05/2021 | G_2_ | 20 | NGS | 1.17 |
| St George21B | 2/03/2021 | -28.1808° | 148.7779° | cotton | 14/07/2021 | G_4_ | 0 | 8.8 | 89.7 | 17/05/2021 | G_2_ | 20 | NGS | 1.13 |
| St George21C | 2/03/2021 | -28.1270° | 148.6673° | mungbean | not tested | | | | | 17/05/2021 | G_2_ | 20 | NGS | 1.27 |
| Griffith21A | 4/03/2021 | -34.5985° | 146.1765° | cotton | 21/07/2021 | G_4_ | 0 | 6.8 | 93 | 22/04/2021 | G_1_ | 20 | NGS | 0.93 |
| Dubbo21A | 5/03/2021 | -32.0231° | 148.1831° | cotton | 21/07/2021 | G_4_ | 3.9 | 2.7 | 100 | 22/04/2021 | G_1_ | 20 | NGS | 0.95 |
| Goondwindi21B | 8/03/2021 | -28.9554° | 150.3922° | cotton | 21/07/2021 | G_4_ | 0 | 10.7 | 86.2 | 22/04/2021 | G_1_ | 20 | NGS | 1.22 |
| Goondiwindi21C | 8/03/2021 | -28.5824° | 150.4409° | cotton | not tested | | | | | 22/04/2021 | G_1_ | 20 | NGS | 1 |
| Hillston21A | 10/03/2021 | -33.3501° | 145.7342° | cotton | 28/07/2021 | G_4_ | 0 | 6.6 | 96.5 | 29/04/2021 | G_1_ | 20 | NGS | 1.43 |
| Moree21A | 18/03/2021 | -29.3841° | 149.9055° | cotton | not tested | | | | | 29/04/2021 | G_1_ | 20 | NGS | 1.16 |
| Moree21B | 18/03/2021 | -29.3905° | 149.9620° | cotton | not tested | | | | | 29/04/2021 | G_1_ | 20 | NGS | 1.44 |
| Moree21C | 18/03/2021 | -29.3889° | 149.7693° | cotton | 28/07/2021 | G_4_ | 0 | 3.8 | 97.5 | 29/04/2021 | G_1_ | 20 | NGS | 1.38 |
| Narrabri21A | 31/03/2021 | -30.1402° | 149.6361° | cotton | not tested | | | | | 17/05/2021 | G_1_ | 20 | NGS | 0.96 |
| Narrabri21B | 31/03/2021 | -30.1566° | 149.5402° | cotton | not tested | | | | | 17/05/2021 | G_1_ | 20 | NGS | 1.17 |
| Narrabri21C | 31/03/2021 | -30.0792° | 149.5225° | cotton | 14/07/2021 | G_3_ | 2.4 | 5.2 | 96.2 | 17/05/2021 | G_1_ | 20 | NGS | 1.1 |

* PB1510R in Hopkinson *et al*., (2020)

# Generation since established in 2007 at Department of Agriculture and Fisheries, Queensland Government, Toowoomba entomology laboratory

‡ Next generation sequencing
